# Implementing a home-based exercise program for patients with advanced, incurable diseases after discharge and their caregivers: lessons we have learned

**DOI:** 10.1186/s13104-015-1523-z

**Published:** 2015-09-30

**Authors:** Waldemar Siemens, Anja Wehrle, Jan Gaertner, Michael Henke, Peter Deibert, Gerhild Becker

**Affiliations:** Department of Palliative Care, University Medical Center Freiburg, Robert-Koch-Straße 3, 79106 Freiburg, Germany; Department of Internal Medicine, Institute for Exercise- and Occupational Medicine, University Medical Center Freiburg, Hugstetter Str. 49, 79106 Freiburg, Germany; Department of Radiation Oncology, University Medical Center Freiburg, Robert-Koch-Straße 3, 79106 Freiburg, Germany

**Keywords:** Palliative care, Palliative medicine, Exercise

## Abstract

**Background:**

Palliative care (PC) patients experience loss of physical function which usually impedes mobility, autonomy and quality of life. We aimed at examining the feasibility of a home-based exercise program for patients with advanced, incurable diseases after discharge.

**Results:**

This was a single-arm pilot study (WHO-ICTRP: DRKS00005048). The 12-week home-based program comprised strength, balance, flexibility and endurance components. Patients with a presumed life expectancy of 6–12 months were recruited during a 6-months period on a specialized PC and a radiation therapy ward. We chose the De Morton Mobility Index as primary outcome. Secondary outcomes were quality of life, 6-min walk test and others. A total of 145 patients were screened, 103 (98 %) out of 105 patients on the specialized PC ward could not be included, mostly because of a low performance status [n = 94; 90 %; Eastern Cooperative Oncology Group (ECOG) >2]. The only two eligible patients declined to participate. Eleven out of 40 patients (28 %) were eligible on the radiation therapy ward. However, only one patient (9 %) participated but dropped out 2 days later (upcoming surgery). Distance to the hospital (n = 3; 30 %) and considering additional tasks as “too much” (n = 3; 30 %) were most common reasons for non-participation.

**Conclusions:**

Establishing a home-based exercise program for inpatients after discharge was not feasible mainly due to non-eligibility and lack of demand. For future trials, we suggest that choosing (1) outpatients with (2) an ECOG of ≤2 and (3) an estimated survival of ≥9 months could enhance participation in home-based exercise programs.

## Background

The decrease in physical functioning impedes quality of life, mobility and autonomy of patients with terminal diseases [1–3]. Physical exercise programs are more and more advocated for patients given palliative care or suffering from advanced cancer in order to address the abovementioned aspects [4–7]. In the hospital, physical exercise is usually supervised by a physiotherapist. To ensure long-term effects of physical exercise, patients might be supported even after their discharge, e.g. by providing a manual with relevant exercises for a home-based exercise program. Interestingly, advanced cancer patients (n = 42, 84 %) stated in a previous survey that they would prefer participating in a physical activity program at home [8]. Some completely home-based exercise programs were recently conducted in patients with advanced cancer of different entities and various life expectancies [9–11]. These studies indicate an improved mobility and a decreased fatigue as a result from physical exercise [9, 11] but also point out challenges in patient recruitment and feasibility [10]. It is necessary to enhance the body of evidence for a comprehensive appraisal of the feasibility and efficacy of such home-based exercise programs.

Therefore, the primary study aim was to examine the feasibility of a home-based exercise program for patients with advanced, incurable diseases after discharge.

## Methods

### Patients

In this interventional single-arm pilot study, adult patients with incurable diseases, a clinician-estimated life expectancy of 6–12 months, an Eastern Cooperative Oncology Group (ECOG) Score ≤2, a numerical rating scale (NRS; 0–10) for pain ≤3 and an adequate cognitive status were included (Table 1). Patients with neurological or orthopedic diseases (that impeded the execution of our home-based exercise program), osseous metastases, heart failure of New York Heart Association (NYHA) stadium III–IV, hypertensive emergency (defined by the American Heart Association as blood pressure that damages organs or exceeds 180 systolic and 120 diastolic) in the last 12 months, bleeding tendency, and dyspnea during movement [verbal rating scale (VRS) ≥2] were excluded to ensure patients’ safety (Table 2). The decision for these inclusion criteria resulted from discussions of our multidisciplinary team (two physicians, two sport and exercise scientists, one psychologist and one theologian) and on the basis of literature [7, 10].

This pilot study was conducted in accordance to the Declaration of Helsinki. It was approved by the Freiburg Ethics Commission and subsequently registered on the World Health Organization International Clinical Trials Registry Platform (WHO-ICTRP: DRKS00005048).

### Intervention

The program was designed to start with two instruction lessons while the patient was still in the hospital. After the patients’ discharge, the 12-week exercise program should be conducted at home with the help of an exercise manual. The home-based exercise program consisted of two parts: a strength training (ca. 35 min; three sets, 10–15 repetitions; five exercises: e.g. squats, wall push-ups) and a combined balance-endurance-flexibility training [ca. 25 min; 15–20 min walking, 5–10 min balance (e.g. tandem, semi-tandem) and flexibility exercises (e.g. pectoral and hip flexor stretch)]. No training equipment was necessary since moderate bodyweight exercises were chosen. Ratings of perceived exertion (RPE) were set between 13 (“somewhat hard”) and 14 points on the Borg RPE scale [12]. The program enabled to vary exercises in a way that an RPE of 13–14 could be theoretically achieved by each patient.

To ensure adherence and intervention fidelity, an exercise manual and a training diary were prepared. Moreover, we planned to call the patients bimonthly to ask if there were problems and barriers during the exercise program.

An extra feature of this intervention was that the patient’s caregiver was asked to participate in the exercise program as caregivers tend to neglect themselves and receive little support by the health care system [13–15]. Moreover, they could contribute to patients’ adherence regarding the home-based program.

### Outcome measures

The primary study aim was to evaluate the feasibility of the home-based exercise program. However, feasibility is a broad concept with up to eight different areas [16]. We focused on the areas *acceptability* (patients’ and staffs’ reaction to study/intervention) and *expansion*. The latter was defined by Bowen et al. as “potential success of an already-successful intervention with a different population or in a different setting” [16]. Since this trial had an exploratory character, acceptability and expansion were only defined qualitatively, not quantitatively.

We planned to measure the following outcome measures at inclusion, after 6 and 12 weeks^1^: primary endpoint was the De Morton Mobility Index (DEMMI) as mobility seems to be a precondition for autonomy which is mostly associated with quality of life [17, 18]. The DEMMI is a validated tool in acute medical population [19] and was considered to be appropriate for palliative care patients.

Secondary outcomes were the European Organization for Research and Treatment of Cancer Quality of Life Core Questionnaire 30 (EORTC QLQ-C30) [20], Romberg test (parallel, semi-tandem and tandem with open eyes), 6-min walk test (6MWT) [21], Barthel Index (BI) [22], five times sit-to-stand test (FTSST) [23], hand grip strength [7] and qualitative interviews (benefits, barriers) after the intervention.

Endpoints for caregivers were the “Indikatoren des Reha-Status 24” (IRES-24) [24], a questionnaire on health status and physical functioning, Romberg test, hand grip strength and 30 s chair [25] stand test.

### Recruitment process

Clinicians on a specialized palliative care ward (August 2013 to January 2014) and on a radiation therapy ward (December 2013 to January 2014) screened patients for eligibility on admission. The patients were hospitalized for symptom control or radiation therapy. Eligible patients were contacted and information on the study was provided. Eligible patients who did not want to participate were asked for reasons whilst emphasizing that non-participation would not result in any disadvantage for the subsequent treatment. No personal health data of patients could be documented if no informed consent was obtained.

### Analysis of results

Numbers and percent were used to present the results. Due to organizational differences during recruitment and different eligibility (mainly ECOG differences) between both wards, each ward was analyzed separately. We aimed at recruiting 25 patients for this pilot study.

## Results

### Specialized palliative care ward

**Fig. 1:**
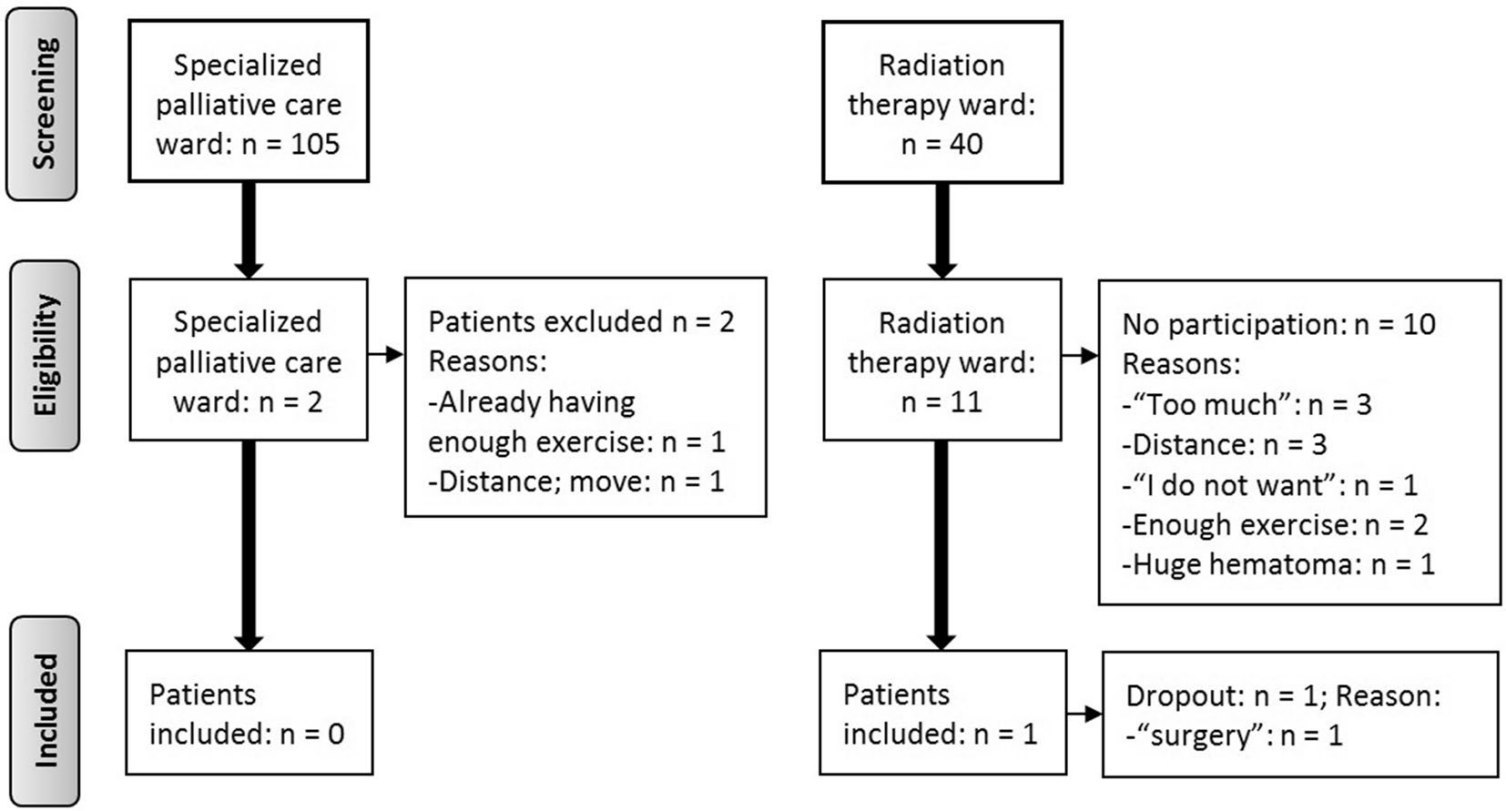
Flow diagram for patients of the specialized palliative care ward and the radiation therapy ward

Two clinicians screened 105 patients on the specialized palliative care ward from August 2013 to January 2014 (Fig. 1). According to the criteria for inclusion and exclusion of our study only two patients (2 %) were eligible. Table 1 shows the number and percentages of patients that fulfilled (column: “yes”) the different inclusion criteria. Eleven patients (10 %) had an ECOG of ≤2 implicating that most patients on the specialized palliative care ward were too fragile for the exercise program. The most prevalent reason for non-participation concerning exclusion criteria was dyspnea with 39 patients (37 %) reaching ≥2 on the VRS (Table 2).

**Table 1:** Inclusion criteria for patients from specialized palliative care ward (n = 105)

	No: n (%)	Yes: n (%)	Total n (%)^a^
≥ 18 years	1 (1)	104 (99)	105 (100)
Life expectancy 6–12 months	64 (61)	38 (36)	102 (97)
ECOG ≤2	94 (90)	11 (10)	105 (100)
Numerical rating scale for pain ≤3	44 (42)	58 (55)	102 (97)
Adequate cognitive status	33 (31)	71 (68)	104 (99)

*n* number of patients, *ECOG* Eastern Cooperative Oncology Group

^a^Total n vary slightly due to missing data

**Table 2:** Exclusion criteria for patients from specialized palliative care ward (n = 105)

	No: n (%)	Yes: n (%)	Total n (%)^a^
Neurological or orthopedic diseases^b^	75 (71)	30 (29)	105 (100)
Osseous metastases	93 (89)	12 (11)	105 (100)
Heart diseases: NYHA III–IV	92 (88)	12 (11)	104 (99)
Hypertensive emergency last 12 months	101 (96)	4 (4)	105 (100)
Bleeding tendency	94 (90)	10 (9)	104 (99)
Dyspnea during movement (VRS ≥2)	66 (63)	39 (37)	105 (100)

*n* number of patients, *NYHA* New York Heart Association, *VRS* verbal rating scale (0–4)

^a^Total n vary slightly due to missing data

^b^Only if disease impeded execution of home-based exercise program

Two patients were eligible but they were not included in the study. The first patient reported having enough exercise by “walking regularly with the dog”. The second patient stated the “distance to the hospital” (60 km) and an “upcoming move” as a main barrier for non-participation.

### Radiation therapy ward

A total of 40 patients were screened for eligibility by three clinicians of the radiation therapy ward from December 2013 to January 2014 (Fig. 1). Twenty-nine patients (73 %) in the radiation therapy ward had an estimated life expectancy over 12 months and, therefore, were not eligible. Eligible patients (n = 11, 28 %; six female and five male; aged 62–86 years, mean: 67.1, standard deviation: 9.4) were contacted by the study investigator. Three out of eleven (27 %) patients did not participate in the exercise program because they felt it would be “too much” in addition to all other burdens. Another three patients (27 %) resigned because of the large distance to the revaluation site (University Medical Center) (see Fig. 1 for additional resaons). Only one (1/11, 9 %) male, 72 years old, patient with lung cancer and his caregiver signed the informed consent but withdrew 2 days later because of the decision for surgery.

## Discussion

### Feasibility

The acceptability for participation on the part of the patients can be considered as low because only one patient out of 11 eligible patients gave informed consent (Fig. 1). However, the decision for not participating is multifactorial and could probably not be summarized in a single reason. It is hardly possible to judge whether the given reason, the complex overall situation, the study design, the intervention, other reasons or a combination of these factors have led to the patients’ decision. The low number of eligible patients can be traced back to our eligibility criteria, which apparently was not appropriate, and the low acceptability may be a consequence of different recruitment barriers (see paragraphs below).

No patient completed the study. Therefore, no judgement can be made on the efficacy of the intervention for this population (expansion) [16].

Feasibility (here: acceptability and expansion) was not defined in a quantitative way because there is a lack of studies with comparable inclusion criteria (especially for life expectancy) [10]. This decision can be criticized. However, an a priori quantitative definition, though arbitrary, is important in order to make a clear and transparent decision.

### Non-eligibility

This pilot study confirms some previously identified recruitment difficulties in palliative care like *low eligibility* and *severe patient illness* [26]. Non-eligibility was the main problem on the specialized palliative care ward (n = 103; 98 %) whereas the acceptability and demand [16] for the home-based exercise program was low for eligible patients (1/11; 9 %) of the radiation therapy ward. In a study by Lowe et al. [10] 524 outpatients were screened, nine (2 %; median survival: 92 days) consented to participate and just three (dropout rate 67 %) completed the 6 week exercise program. It is suggested that patients with a better performance status and longer (median) survival clearly contribute to a study’s feasibility as seen from the example of Cheville et al. [93 patients screened, 66 (71 %) randomized; dropout rate: 7/33; 21 %] [9].

### Recruitment barriers

We tried to recruit *inpatients* for a home-based program after their discharge. It is noticeable that (especially palliative care) inpatients experience probably more burden by symptoms, psychosocial problems and are more confronted with diagnostic and therapeutic interventions than *outpatients* that were recruited in two comparable studies [9, 10]. In addition, being not at home and spending thoughts (i.e. cognitive capacity) on further treatments or different psychosocial questions could have contributed to the low participation of eligible patients especially from the radiation therapy ward.

Eligible patients on the radiation therapy ward were possibly confused by the term *“palliative”* as conversations about patients’ prognosis are often neglected [27]. Moreover, advanced cancer patients are often not aware that their situation or treatment is non-curative [28, 29].

Lowe’s [10] and our recruitment difficulties are in contrast to an interviewer-administered needs assessment [8] where 39 of 50 terminally ill patients (78 %) stated to be interested in a physical activity program. *Social desirability bias* may have led to the overoptimistic survey results [30, 31].

### Implications and suggestions for future research

Acceptability, demand (radiation therapy ward) and especially non-eligibility (specialized palliative care ward) were the main problems for non-participation in this study [16]. Several studies show that these problems can be reduced by using wider limits (than in this study) with respect to life expectancy or performance status [4, 7, 32–34].

A reasonable combination of *modulating factors* (Table 3) could contribute to a higher demand or feasibility for home-based exercise programs. However, emerging costs and practicability should be taken into account to enable transfer in daily clinical practice [16].

**Table 3:** Modulating factors for future study designs

Category	Modulating factors
Patients	Clinician-estimated life expectancy ECOG Dyspnea In-versus outpatients If inpatients: involvement of one or more wards Amount of exercise experience
Intervention	Variable exercises: ensure similar RPE Lying, sitting or standing exercises Duration period Length of an exercise Exercises per week
Supportive means	Phone calls Diary Manual Supervision by exercise professional/physiotherapist Material: elastic bands etc. Instruction video Cooperation of staff in another facility (e.g. hospice) Inclusion of caregiver

*ECOG* Eastern Cooperative Oncology Group

Based on our experience from this trial and on two comparable studies [9, 10], we suggest that the following criteria could enhance participation and enable evaluation of the benefits of a home-based exercise program:

- palliative care outpatients
- ECOG of ≤2
- estimated survival >9 months

## Conclusion

Implementing a home-based exercise program was not feasible for patients with advanced, incurable diseases after discharge from a specialized palliative care ward and a radiation therapy ward. Patients on specialist palliative care and radiotherapy wards might be too sick and burdened by other symptoms and medical interventions to feel comfortable in engaging themselves in a home-based exercise program.

## Abbreviations

BI: Barthel Index
DEMMI: De Morton Mobility Index
ECOG: Eastern Cooperative Oncology Group
EORTC QLQ-C30: European Organization for Research and Treatment of Cancer Quality of Life Core Questionnaire 30
FTSST: five times sit-to-stand test
IRES-24: Indikatoren des Reha-Status 24
PC: palliative care
RPE: ratings of perceived exertion
VRS: verbal rating scale
WHO-ICTRP: World Health Organization International Clinical Trials Registry Platform
6MWT: 6-min walk test

## Footnotes

1 As a consequence of the non-feasibility of this study and for the upcoming future trial we slightly changed the study duration to six weeks and chose a interim measurement of 3 weeks (http://apps.who.int/trialsearch/Trial2.aspx?TrialID=DRKS00005048; http://drks-neu.uniklinik-freiburg.de/drks_web/navigate.do?navigationId=trial.HTML&TRIAL_ID=DRKS00005048).
